# Baicalein Induces G_2_/M Cell Cycle Arrest Associated with ROS Generation and CHK2 Activation in Highly Invasive Human Ovarian Cancer Cells

**DOI:** 10.3390/molecules28031039

**Published:** 2023-01-20

**Authors:** Tzu-Chao Chuang, Wei-Syun Shao, Shih-Chung Hsu, Shou-Lun Lee, Ming-Ching Kao, Vinchi Wang

**Affiliations:** 1 Department of Chemistry, Tamkang University, New Taipei 251301, Taiwan; 2 Department of Early Childhood Care and Education, University of Kang Ning, Taipei 114311, Taiwan; 3 Department of Biological Science and Technology, China Medical University, Taichung 406040, Taiwan; 4 Department of Neurology, Cardinal Tien Hospital, New Taipei 231009, Taiwan; 5 School of Medicine, College of Medicine, Fu-Jen Catholic University, New Taipei 242062, Taiwan

**Keywords:** baicalein, CHK2, DNA damage response, flavone, ovarian cancer, pro-oxidant

## Abstract

Ovarian cancer is a lethal gynecological cancer because drug resistance often results in treatment failure. The CHK2, a tumor suppressor, is considered to be an important molecular target in ovarian cancer due to its role in DNA repair. Dysfunctional CHK2 impairs DNA damage-induced checkpoints, reduces apoptosis, and confers resistance to chemotherapeutic drugs and radiation therapy in ovarian cancer cells. This provides a basis for finding new effective agents targeting CHK2 upregulation or activation to treat or prevent the progression of advanced ovarian cancer. Here, the results show that baicalein (5,6,7-trihydroxyflavone) treatment inhibits the growth of highly invasive ovarian cancer cells, and that baicalein-induced growth inhibition is mediated by the cell cycle arrest in the G_2_/M phase. Baicalein-induced G_2_/M phase arrest is associated with an increased reactive oxygen species (ROS) production, DNA damage, and CHK2 upregulation and activation. Thus, baicalein modulates the expression of DNA damage response proteins and G_2_/M phase regulatory molecules. Blockade of CHK2 activation by CHK2 inhibitors protects cells from baicalein-mediated G_2_/M cell cycle arrest. All the results suggest that baicalein has another novel growth inhibitory effect on highly invasive ovarian cancer cells, which is partly related to G_2_/M cell cycle arrest through the ROS-mediated DNA breakage damage and CHK2 activation. Collectively, our findings provide a molecular basis for the potential of baicalein as an adjuvant therapeutic agent in the treatment of metastatic ovarian cancer.

## 1. Introduction

Ovarian cancer is one of the most aggressive and leading causes of cancer death among female cancers worldwide, with an estimated 200,000 deaths in 2020 [1]. In 2021, more than 22,000 ovarian cancer deaths occurred in the U.S., the fifth leading cause of cancer-related death in women [2]. The high-grade serous ovarian cancer is the most prevalent type of ovarian cancer and is the leading cause of death from all forms of ovarian cancer due to its highly aggressive clinical course [3]. The activation of oncogenes such as K-Ras and ErbB2 and the inactivation or mutation of tumor suppressor genes such as BRCA-1 and TP53 are frequently associated with ovarian cancer [4,5,6]. Because ovarian cancer is asymptomatic in its early stage, most ovarian cancer patients are diagnosed at an advanced stage, when the cancer cells have metastasized, usually to the peritoneum or pelvis, resulting in a poor 5-year survival rate for patients. Therefore, there remains a need for new approaches to target highly aggressive ovarian cancer at the molecular level.

DNA damage affects the interpretation and transmission of genetic information. Upon DNA damage, DNA damage response (DDR) networks, including DNA damage recognition, checkpoint activation, cell cycle arrest, DNA repair, apoptosis, and immune clearance, are activated [7]. Once DNA double strand breaks (DSBs) occur, cells must suspend all of the cell cycle progression for DNA repair, and the MRN (Mre11/Rad50/Nbs1) complex is recruited to DNA damage sites by histone γH2AX and RAD17 to initiate DNA repair. The MRN complex on DSBs recruits and directly binds to the inactive homodimeric ATM protein kinase and converts it into an active monomer [8,9]. Activated ATM phosphorylates Thr68 and other serine residues on CHK2 (checkpoint kinase 2) to fully activate CHK2 to prevent cell cycle progression, thereby allowing DSB repair. Activated CHK2 phosphorylates many intracellular targets; for example, CHK2 phosphorylates and inactivates CDC25A and CDC25C phosphatases. Activated CDC25A dephosphorylates and activates CDK2, thereby promoting S phase entry and progression, while activated CDC25C dephosphorylates and activates CDK1 to promote G_2_/M phase progression [9]. Moreover, activated ATM also phosphorylates H2AX (Ser139) in the chromatin surrounding DSBs, so forming the γH2AX foci. After the H2AX phosphorylation, chromatin remodeling complexes and DNA repair proteins are recruited to DSB sites for DNA repair [7,9,10]. In addition, activated ATM and CHK2 also phosphorylate and activate p53 on Ser15 and Ser20, respectively, and activated p53 induces G_1_ cell cycle arrest by upregulating p21. However, under sustained cellular injury, p53 activation induces apoptosis through the transcription of proapoptotic genes such as PUMA and NOXA [9].

Epidemiological studies and systematic analyses suggest that increased dietary flavonoid intake is associated with a reduced risk of some cancers; however, results on the impact of flavonoid intake on particular cancers remain inconclusive. The inconsistent findings of chemopreventive activity may be due to different flavonoids and different cancer sites [11,12]. Baicalein (5,6,7-trihydroxyflavone) is a flavone derived from the root of *Scutellaria baicalensis* Georgi that has long been used as a traditional anti-inflammatory medicine. Baicalein has various properties, such as being an antioxidant, anti-inflammatory, antimicrobial, anticancer and neuroprotective [13,14]. Regarding the anticancer activity of baicalein, studies have shown that baicalein engages in antiproliferative activity against a variety of different cancers by acting on various biological processes, including cell proliferation, apoptosis, cell cycle, metastasis and epithelial-mesenchymal transition (EMT). Baicalein has been shown to inhibit cancer growth by inhibiting oncogenic molecules such as Akt and MAPK, inducing apoptosis of cancer cells by regulating apoptosis-related proteins such as Bcl-2 and Bax, and causing cell cycle arrest by modulating cell cycle regulatory proteins such as cyclins and cyclin-dependent kinases (CDKs) [15]. Baicalein also inhibits pathological EMT action and matrix metalloproteinases (MMPs) activity to attenuate metastasis [16,17]. Furthermore, recent studies have shown that baicalein-induced apoptosis in human lung, bladder, and breast cancer cells is associated with the production of reactive oxygen species (ROS) [18,19,20].

ROS are recognized as signaling molecules that regulate different physiological functions such as cell proliferation, differentiation, and immune responses. The production of ROS can activate pro-tumorigenic signaling to enhance cell survival; conversely, increased ROS can also promote antitumorigenic signaling, which triggers oxidative stress-induced cell death. Cancer cells can detoxify elevated ROS levels by expressing elevated levels of antioxidant proteins, thereby establishing altered redox balance, maintaining pro-tumorigenic signaling and resisting apoptosis [21]. Flavonoids are generally considered scavengers of ROS, including hydroxyl radicals and superoxide anions, and this antioxidant activity is thought to potentially help prevent cancer. Some flavonoids have shown to have dual roles in ROS homeostasis; they act as antioxidants under normal conditions to eliminate pro-tumorigenic signals and are potent pro-oxidants in cancer cells to trigger the apoptotic signals [22]. For example, flavonoids react with superoxide anions to form relatively stable aryloxy radicals on flavonoids through single electron transfer so they can scavenge superoxide anions [23]. The pyrogallol moiety of flavonoids also contributes to superoxide radical scavenging activity [24]. In contrast, some flavonoids, such as baicalein and quercetin, unexpectedly increase the production of hydroxyl radicals in the Fenton system [25]. Therefore, certain flavonoids may act as antioxidants and pro-oxidants, depending on the concentration and source of free radicals and cellular conditions.

This study explores another possible mechanism to elucidate the growth-inhibitory effect of baicalein on aggressive human ovarian cancer. 

## 2. Results

### 2.1. Baicalein Suppresses Highly Invasive Ovarian Cancer Cell Proliferation by Inducing G_2_/M Cell Cycle Arrest

**Figure 1:**
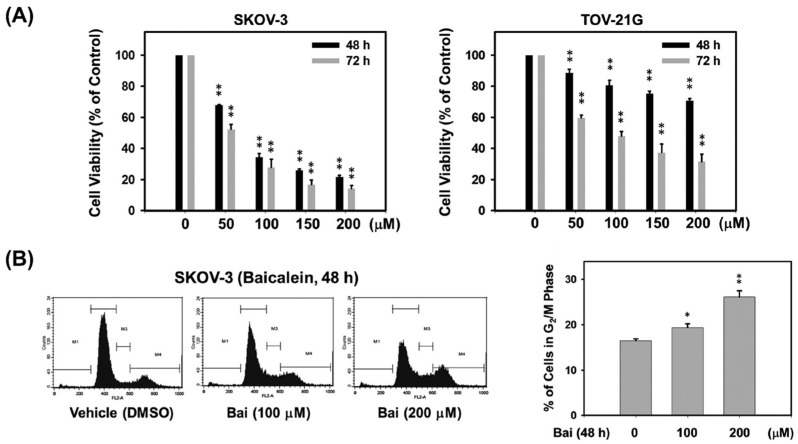
Effect of baicalein on the proliferation of highly invasive human ovarian cancer cells. (**A**) Anti-proliferative effect of baicalein on ErbB2-positive SKOV-3 (ErbB2^high^) and TOV-21G (ErbB2^low^) ovarian cancer cells. Cells were treated with vehicle control (0.15% DMSO, *v*/*v*) and different concentrations (50–200 μM) of baicalein for 48 and 72 h, and the anti-proliferative effect was evaluated by MTT analysis. The number of viable cells is expressed as a percentage of the vehicle control. (**B**) Cell cycle profiles of SKOV-3 cells treated with 100 or 200 μM baicalein for 48 h were analyzed by flow cytometry. Histograms depicting cell cycle distribution are expressed as the proportion of SKOV-3 cells accumulating in the G_2_/M phase after treatment with 100 or 200 μM baicalein for 48 h. All data are expressed as the mean ± SE of three independent experiments. *, *p* < 0.05; **, *p* < 0.01, compared with the vehicle group.

To elucidate whether baicalein inhibits the growth of aggressive ovarian cancer cells, we first assessed the cell viability of highly invasive human SKOV-3 and TOV-21G ovarian cancers with different ErbB2 levels treated with baicalein. As shown in Figure 1A, treatment of SKOV-3 (ErbB2^high^) and TOV-21G (ErbB2^low^) cells with 50–200 μM baicalein for 48 and 72 h produced significant dose- and time-dependent growth inhibition. The IC_50_ values of baicalein treatment for 72 h on SKOV-3 and TOV-21G cells were approximately 54 and 91 μM, respectively. These results show the growth-inhibitory effect of baicalein on ovarian cancer cells, especially in cells overexpressing ErbB2. Therefore, next, to verify whether the growth-inhibitory effect of baicalein was derived from an effect on cell cycle progression, the cell cycle distribution of SKOV-3 cells exposed to baicalein was assessed by PI staining and analyzed by flow cytometry. The results showed that baicalein treatment resulted in a significant accumulation of cells in G_2_/M phase in SKOV-3 cells (Figure 1B). Cells treated with 200 μM baicalein for 48 h exhibited more pronounced G_2_/M arrest, with a 1.6-fold higher percentage of G_2_/M phase than vehicle controls.

### 2.2. Baicalein Causes ROS Generation and DNA Breakage in Ovarian Cancer Cells

**Figure 2:**
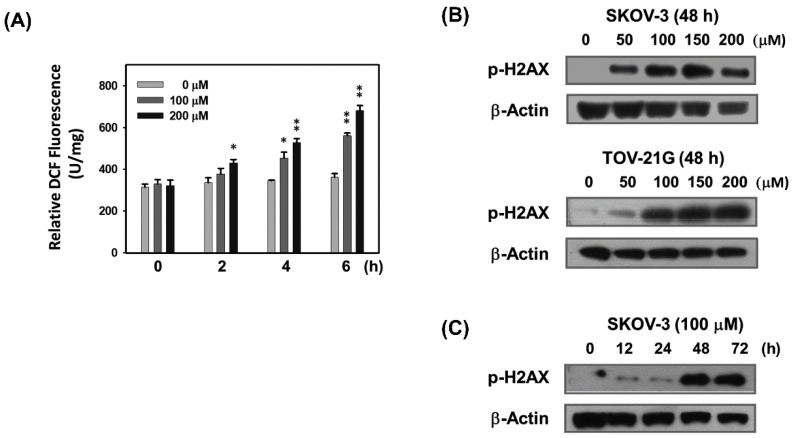
Baicalein causes ROS generation and DNA damage in ovarian cancer cells. (**A**) Baicalein treatment results in ROS production. SKOV-3 cells were pretreated with H2DCFDA for 1 h and then treated with 100 or 200 μM baicalein for 2–6 h, and the fluorescence intensity due to ROS production was measured. (**B**,**C**) Baicalein treatment causes DNA double-strand breaks. (**B**) SKOV-3 and TOV-21G cells were treated with baicalein (50–200 μM) for 48 h or (**C**) SKOV-3 cells were treated with baicalein (100 μM) for the indicated times (12–72 h), and the protein levels of phosphorylated H2AX (a DNA double-strand break marker) were measured by immunoblot analysis. Significance was defined as *, *p* < 0.05 and **, *p* < 0.01 relative to the vehicle control.

Based on the results presented above, it is speculated that the growth-inhibitory effect of baicalein on ovarian cancer cells is due to the perturbation of cell cycle checkpoints. Cell cycle arrest is typically initiated by checkpoint activation in response to DNA damage. ROS have been reported to be involved in DNA damage leading to cell cycle arrest and/or apoptosis [5,7]. Therefore, we next tested whether baicalein treatment causes ovarian cancer cells to generate ROS and cause DNA damage. As shown in Figure 2A, the results indicated that baicalein treatment produced significant ROS in a dose- and time-dependent manner in SKOV-3 cells. Furthermore, immunoblotting (Western blotting) data showed that baicalein treatment increased the phosphorylation of H2AX (γH2AX) at Ser139, a marker for DNA double-strand breaks (DSBs), in a dose-dependent manner in SKOV-3 and TOV-21G cells (Figure 2B). Baicalein also time-dependently increased the γH2AX in SKOV-3 cells (Figure 2C). These data suggest that ROS may be involved in baicalein-mediated DNA damage in ovarian cancer cells.

### 2.3. Baicalein Activates the ATM/CHK2/CDC25C Signaling Pathway in Ovarian Cancer Cells

**Figure 3:**
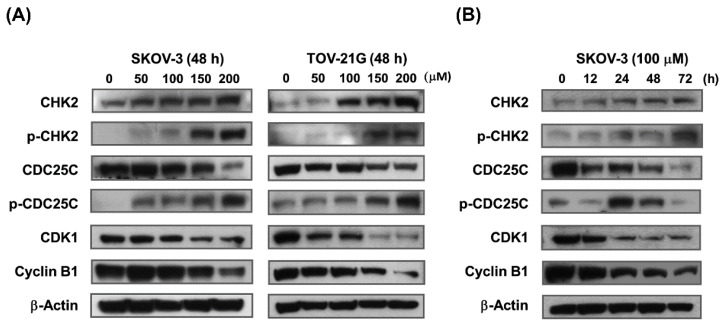
Baicalein modulates cell cycle regulatory proteins in G_2_/M phase via the ATM/CHK2/CDC25C signaling axis in ovarian cancer cells. (**A**) SKOV-3 and TOV-21G cells were treated with 50–200 μM baicalein for 48 h. (**B**) SKOV-3 cells were treated with 100 μM baicalein for 12–72 h. Immunoblotting showed the effect of baicalein on the protein levels of phospho-CHK2 and phospho-CDC25C, as well as the protein levels of CHK2, CDC25C, CDK1 and cyclin B1. β-actin was used as a loading control.

To elucidate the molecular mechanisms controlling baicalein-mediated cell cycle arrest in the G_2_/M phase, immunoblot assay was performed on control and treated cells to evaluate the effect of baicalein on G_2_/M phase cell cycle regulatory proteins such as CHK2, CDC25C, CDK1 and cyclin B1. The results showed that baicalein treatment had a significant dose-dependent effect on the upregulation of CHK2 and the phosphorylation of CHK2 and CDC25C and the downregulation of CDC25C, CDK1 and cyclin B1 in SKOV-3 and TOV-21G cells (Figure 3A). Likewise, baicalein also significantly affected the expression of key regulatory proteins associated with the G_2_/M cell cycle checkpoint in SKOV-3 cells in a time-dependent manner (Figure 3B). The results showed that baicalein induces G_2_/M arrest by modulating G_2_/M cell cycle regulatory proteins in ovarian cancer cells.

### 2.4. Blocking of CHK2 Activity Attenuates Baicalein-Induced G_2_/M Arrest in Ovarian Cancer Cells

**Figure 4:**
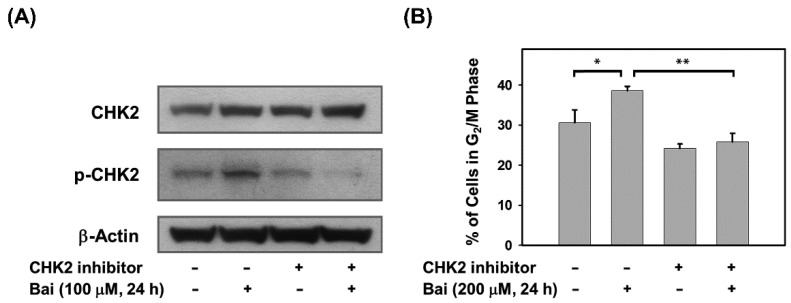
Pharmacological inhibition of CHK2 blocks baicalein-mediated G_2_/M cell cycle arrest. (**A**) SKOV-3 cells were pretreated with 30 μM CHK2 inhibitor (Chk2 inhibitor II) for 2 h and then with 100 μM baicalein for 24 h. The protein levels of phosphorylated CHK2 (Thr68) and total CHK2 in cells after treatment were measured by immunoblot analysis. (**B**) SKOV-3 cells were pretreated with 30 μM CHK2 inhibitor (Chk2 inhibitor II) for 2 h and then treated with 200 μM baicalein for 24 h. The percentage of cells accumulated in the G_2_/M phase after treatment was assessed by flow cytometry. Significance was defined as *, *p* < 0.05 and **, *p* < 0.01.

Because baicalein significantly activates the ATM/CHK2/CDC25C signaling axis, which may lead to G_2_/M arrest, we next determined whether blocking CHK2 could attenuate baicalein-induced G_2_/M arrest to confirm the role of CHK2 activation in baicalein-mediated G_2_/M arrest in ovarian cancer cells. SKOV-3 cells were pretreated with a CHK2 inhibitor (Chk2 inhibitor II) for 2 h and then incubated with baicalein for 24 h. The data showed that the CHK2 inhibitor indeed reduced the extent of CHK2 phosphorylation (Figure 4A) and protected SKOV-3 cells from baicalein-mediated G_2_/M arrest (Figure 4B). These data illustrate the critical role of CHK2 in baicalein-mediated G_2_/M cell cycle arrest in ovarian cancer cells.

## 3. Discussion

**Figure 5:**
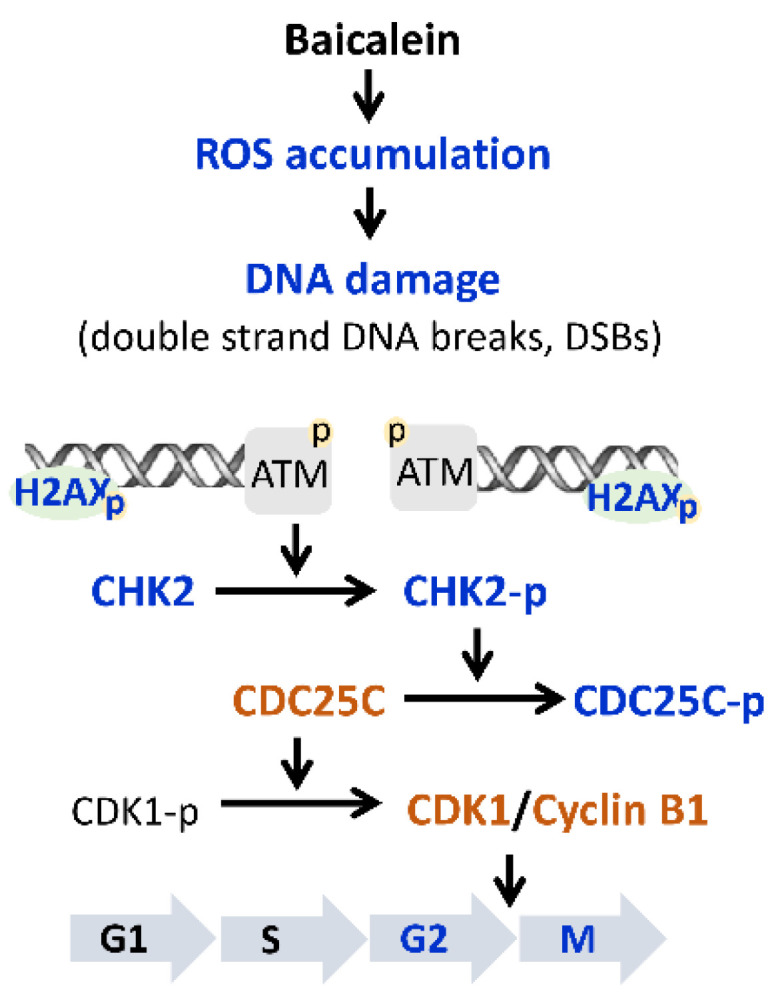
Schematic illustration of baicalein-induced G_2_/M cell cycle arrest in ErbB2-positive ovarian cancer cells via activation of the ATM/CHK2/CDC25C signaling axis. Treatment of ovarian cancer cells with baicalein induces ROS generation, causes DNA double-strand breaks, and triggers the DNA damage response to ATM signaling to arrest cell cycle progression at the G_2_/M phase. Blue font represents an increase, and orange font represents a decrease.

Baicalein exerts its anticancer effects in different cancer cells, targeting multiple biological processes, such as cell proliferation, apoptosis, autophagy, metastasis, cell cycle and epithelial-mesenchymal transition [15]. All these results suggest that baicalein exerts different anticancer effects on the proliferation and survival of different types or properties of cancer cells. For example, baicalein is known to inhibit the expression of VEGF, HIF-1α, c-Myc or NF-κB in ovarian cancer cells, thereby inhibiting the metastatic potential of ovarian cancer cells, while the effect on normal cells is generally less [15,26]. In this study, our findings demonstrate that baicalein treatment inhibits the viability of highly invasive human ovarian cancer cells, and that this growth inhibitory effect is partly related to ROS generation. Accumulated ROS molecules are associated with the induction of double-strand DNA breaks (DSBs) and the activation of CHK2, thereby triggering G_2_/M cell cycle arrest (Figure 5). To our knowledge, this study identifies CHK2 as the key molecular target of baicalein in human ovarian cancer cells for the first time.

The overexpression of EGFR and/or ErbB2 frequently occur in human ovarian cancer; this is associated with more aggressive tumor behavior and poorer patient outcomes. In a previous study, we found that baicalein inhibited ErbB2-mediated malignant transformation of ErbB2-overexpressing ovarian cancer cells by downregulating ErbB2 gene expression at the transcriptional level (unpublished results). In Figure 1A, the data show that the growth-inhibitory effect of baicalein on SKOV-3 (high ErbB2 expression) cells is higher than that of TOV-21G (low ErbB2 expression) cells. Based on these findings, we speculate that the growth-inhibitory effect of baicalein on SKOV-3 cells is better than that on TOV-21G cells, because baicalein can not only upregulate and activate CHK2 to cause G_2_/M cell cycle arrest, but also inhibit the expression of ErbB2 to inhibit cell proliferation.

There is evidence that certain chemotherapeutic drugs are selectively toxic to cancer cells, as human cancer cells appear to produce ROS at a much higher rate than normal cells, triggering oxidative stress-induced cell death. These chemotherapy drugs push these already oxidatively stressed cancer cells beyond the cellular limits, leading to further cellular damage [18]. Baicalein can not only scavenge free radicals in vitro, such as DPPH (1,1-diphenyl-2-picrohydrazine) radicals, superoxide radicals, hydroxyl radicals and alkyl radicals but also effectively reduce the damage of cellular oxidative stress induced by lipopolysaccharide, H_2_O_2_ or UV in macrophages, Schwann cells or skin cells [23,27,28,29,30]. On the other hand, baicalein may trigger apoptotic cell death through intracellular ROS generation and accumulation in bladder and lung cancer cells, suggesting that baicalein may act as a pro-oxidant to induce caspase-dependent apoptosis [18,19]. Chou et al. (2007) identified the free radicals formed by certain flavonoids in several intact cellular systems and showed that the pro-oxidative effect of baicalein to generate hydroxyl radicals may be through inhibition of 12-lipoxygenase (12-LOX) in human platelet suspension [23]. It is known that 12-LOX is involved in the regulation of ovarian cancer cell growth and survival, and the expression level of 12-LOX tends to be significantly elevated in ovarian cancers (such as the SKOV-3 cells) compared with normal ovarian epithelial cells [31,32]. Therefore, the pro-oxidative effect of baicalein, a specific inhibitor of 12-LOX, on ovarian cancer cells may occur through inhibiting the activity of 12-LOX, thereby inducing the accumulation of hydroxyl radicals. However, more studies are needed to demonstrate the association of the pro-oxidative effects of baicalein and 12-LOX in causing G_2_/M arrest in ovarian cancer cells.

Following the DNA damage, highly coordinated DNA damage response signals, mainly including ATM- and ATR-mediated signaling pathways, are activated to recognize DNA breaks and block cell cycle progression to promote DNA repair; alternatively, in extensive or irreparable DNA-damaged cells, apoptosis is induced by p53 [9,10]. In addition to our observation that baicalein treatment increased intracellular ROS and DNA double-strand break levels in ovarian cancer cells resulting in cell arrest, we also found that baicalein induced apoptosis in TOV-21G cells, but not in SKOV-3 cells. TOV-21G cells are p53 wild-type cells; however, SKOV-3 cells lack p53 protein and transcript expression due to a single nucleotide deletion in exon 4 [33]. Therefore, the ATM/p53 signaling axis may be involved in baicalein-induced apoptosis of TOV-21G cells, and baicalein-induced G_2_/M phase arrest of SKOV-3 cells is independent of the ATM/p53/p21 signaling axis.

In conclusion, this study revealed a novel chemotherapeutic effect of baicalein on highly invasive ErbB2-positive ovarian cancer cells. The results of this study suggest that one of the baicalein-mediated cytostatic effects is due to DNA damage (especially double-strand DNA breaks) and activation of CHK2, which is caused in part by the production of ROS.

## 4. Materials and Methods

### 4.1. Cell Culture and Chemicals 

All ovarian cancer cell lines used were obtained from the American Type Culture Collection (Manassas, VA, USA). SKOV-3 human ovarian cancer cells were cultured in McCoy’s 5A medium supplemented with 10% FBS (fetal bovine serum) and TOV-21G human ovarian cancer cells were cultured in a 1:1 mixture of MCDB 105 Medium plus Medium 199 supplemented with 15% FBS. All cells were grown at 37 °C in a humidified incubator with 5% CO_2_. Baicalein (5,6,7-trihydroxyflavone) was purchased from Alfa Aesar (Tewksbury, MA, USA). Chk2 inhibitor II (2-(4-(4-Chlorophenoxy)phenyl)-1*H*-benzimidazole-5-carboxamide hydrate), DMSO (dimethyl sulfoxide), McCoy’s 5A medium, MCDB 105 Medium, MTT (3-(4,5-Dimethylthiazol-2-yl)-2,5-diphenyltetrazolium bromide) and NAC (*N*-acetyl cysteine) were purchased from Sigma-Aldrich (St. Louis, MO, USA). FBS, H2DCFDA (2′-7′-dichlorofluorescin diacetate), Medium 199 were purchased from Invitrogen (Carlsbad, CA, USA). 

### 4.2. Cell Viability Assay

Cells (5000/well) were seeded in 96-well plates for 24 h and treated with baicalein or vehicle (DMSO) at the indicated concentrations, and then incubated for 48 and 72 h. Following incubation, the media were discarded and the cell viability was measured by the MTT cell viability assay. Viable cells were stained with 200 μL of MTT solution [5 mg/mL in 1X PBS buffer (pH 7.4; 137 mM NaCl, 2.7 mM KCl, 10 mM Na_2_HPO_4_, 1.8 mM KH_2_PO_4_)] at 37 °C for 5 h. After incubation, the MTT solution was removed, 200 μL of DMSO was added to each well, and the plates were shaken for 30 min to lyse MTT-formazan formed by metabolically viable cells. Absorbance was measured at 630 nm (MRX Revelation, Thermo Labsystems, Waltham, MA, USA).

### 4.3. Flow Cytometric Analysis

The effect of baicalein on cell cycle distribution was assessed by flow cytometry after staining the cells with propidium iodide (PI). In brief, after baicalein treatment, cells were fixed with ice-cold 70% ethanol overnight at 4 °C. Prior to analysis, the cells were washed twice with PBS buffer and then incubated with PI solution (50 μg/mL PI in PBS with 1% Tween-20 and 10 μg RNase) for 1 h at 4 °C in the dark. Stained cells were analyzed with a BD FACSCalibur Instrument (BD Biosciences, San Jose, CA, USA). Cell cycle distribution data were analyzed using CellQuest (v. 7.5.3) software (BD Biosciences, San Jose, CA, USA).

### 4.4. Determination of ROS Generation

Cells (1 × 10^6^) were seeded in a 100 mm petri dish and allowed to attach overnight. The next day, the medium was completely removed, and the cells were washed twice with PBS and incubated with 10 μM H2DCFDA in serum-free medium for 1 h. H2DCFDA was then removed, and the cells were washed twice with PBS. These washed cells were treated with DMSO (vehicle) or 100 and 200 μM baicalein and incubated for 2, 4 and 6 h. Baicalein-treated cells and control cells were collected and lysed with RIPA lysis buffer. After cell lysis, the extract was centrifuged at 13,000× *g* for 10 min at 4 °C to collect the supernatant. Fluorescence readings of DCF (2′-7′-dichlorofluorescein) in the supernatant were measured using a Hitachi F-2500 fluorescence spectrophotometer (Hitachi High Technologies America, Schaumburg, IL, USA) at an excitation wavelength of 485 nm and an emission wavelength of 535 nm. The protein content of the supernatant was measured using a Bio-Rad protein assay kit (Bio-Rad, Hercules, CA, USA). The relative fluorescence units of DCF were measured and normalized to the protein content of the cell lysates.

### 4.5. Immunoblot Analysis

Immunoblotting (Western blotting) was performed as previously described in Chuang et al. [34]. Antibodies against phospho-CHK2 (Thr-68), phospho-CDC25C (Ser-216), phospho-H2AX (Ser-139) were obtained from Epitomics (Burlingame, CA, USA). Antibodies against CDC25C, CDK1 and Cyclin B1 were obtained from Millipore (Burlington, MA, USA). Antibody against CHK2 was obtained from BD Bioscience (Franklin Lakes, NJ, USA). The antibody to β-actin was from Novus Biologicals (Centennial, CO, USA). HRP-conjugated secondary antibody was from PerkinElmer Life Sciences (Boston, MA, USA).

### 4.6. Statistical Analysis

The data are shown as the mean ± SEM of three independent experiments. Comparisons were performed by Student’s *t*-test. Significance was defined as *, *p* < 0.05 and **, *p* < 0.01 relative to the vehicle control.

## Data Availability

The data presented in this study are available in the main text of this article.

## Abbreviations

ATM, ataxia telangiectasia mutated kinase; CDC25C, cell division cycle 25C; CDK, cyclin-dependent kinase; CHK2, checkpoint kinase 2; DDR, DNA damage response; DSBs, double-strand DNA breaks; H2AX, H2A histone family member X; ROS, reactive oxygen species.
